# Presence of N-glycosylated transthyretin in plasma of V30M carriers in familial amyloidotic polyneuropathy: an escape from ERAD

**DOI:** 10.1111/jcmm.12024

**Published:** 2013-02-07

**Authors:** Anabela C Teixeira, Maria J Saraiva

**Affiliations:** a Molecular Neurobiology, Instituto de Biologia Molecular e Celular, IBMCPorto, Portugal; b Instituto de Ciências Biomédicas de Abel Salazar, ICBAS, University of PortoPortugal

**Keywords:** Transthyretin, N- glycosylation, ERAD

## Abstract

Familial amyloid polyneuropathy (FAP) is an autosomal dominant disease characterized by deposition of amyloid related to the presence of mutations in the transthyretin (TTR) gene. TTR is mainly synthesized in liver, choroid plexuses of brain and pancreas and secreted to plasma and cerebrospinal fluid (CSF). Although it possesses a sequon for N-glycosylation N-D-S at position 98, it is not secreted as a glycoprotein. The most common FAP-associated mutation is TTR V30M. In a screening for monoclonal antibodies developed against an amyloidogenic TTR form, we detected a distinct TTR with slower electrophoretic mobility in Western of plasma from carriers of the V30M mutation, not present in normal plasma. Mass spectrometry analyses of this slower migrating TTR (SMT) identified both wild-type and mutant V30M; SMT was undetectable upon N-glycosidase F treatment. Furthermore, SMT readily disappeared in the plasma of V30M - FAP patients after liver transplantation and appeared in plasma of transplanted domino individuals that received a V30M liver. SMT was also detected in plasma, but not in CSF of transgenic mice for the human V30M mutation. A hepatoma cell line transduced to express human V30M did not present the SMT modification in secretion media. Glycosylated TTR was absent in fibrils extracted from human kidney V30M autopsy tissue or in TTR aggregates extracted from the intestine of human TTR transgenic mice. Studies on the metabolism of this novel, glycosylated TTR secreted from FAP liver are warranted to provide new mechanisms in protein quality control and etiopathogenesis of the disease.

## Introduction

Familial amyloidotic polyneuropathy (FAP) is characterized by extracellular amyloid deposition, in particular, in the peripheral nervous system. Deposits are mainly composed by mutated transthyretin (TTR) being V30M the most common mutation associated with FAP. More than 100 TTR mutations have been described, the great majority associated with FAP. The vast majority of FAP patients are heterozygous for the mutant TTR. Some forms are not neuropathic, but rather cardiomyopathic whereas other variants are not pathogenic (for a tabulation see: http://amyloidosismutations.com/attr.html).

TTR is a secreted non-glycosylated tetrameric protein of 55 KD that is mainly synthesized in liver whose major function is the plasma transport of thyroxine (T_4_) and retinol-binding protein (RBP). Native TTR comprises four identical subunits each containing 127 amino acid residues with a molecular mass of approximately 14 KD. The mechanism of extracellular fibrillogenesis is not fully understood, but several studies point out that amyloidogenic TTR mutants influence conformational changes that induce tetramers dissociation into partially unfolded monomers which self-assemble into amyloid fibrils. (for a review see [1]).

Although no evidence exists for the circulation of TTR aggregates in plasma from FAP patients [2], recent biophysical studies on TTR L55P, associated with a very aggressive form of FAP, revealed significative conformational changes as compared with the wild-type protein or other amyloidogenic clinically less aggressive mutations [3]. Thus, the three-dimensional studies of the L55P variant indicated that the OH group of tyrosine 78 plays an important role in maintaining the tertiary structure of the AB loop. Based on these findings, a specific mutation was designed to replace tyrosine for phenylalanine [4]. Biochemical characterization of Y78F showed that this variant adopts a tetrameric conformation as normal TTR and retains the ability to bind T_4_, indicating a functional tetrameric structure. Under acidic pH, the tyr—phe substitution at position 78 is more prone to form fibrils as compared with non-mutated TTR. It was hypothesized that Y78F exhibits the characteristics of an intermediate structure in the fibrillogenesis pathway and might represent an early event in TTR amyloidogenesis. Interestingly, this mutation designed *in silico* was found associated with peripheral neuropathy, carpal tunnel syndrome and skin amyloidosis [5].

Monoclonal antibodies (mabs) produced in mice against highly aggressive amyloidogenic synthetic TTR mutants were shown to react with high molecular weight TTR aggregates, but do not recognize soluble native TTR when tested under ELISA (enzyme-linked immunoassay). It was hypothesized that these mabs recognize cryptic epitopes that are exposed in mutant TTRs resembling aggregated TTR [6]. Interestingly, these mabs, under specific conditions, reacted with TTR from plasma of FAP patients and/or asymptomatic carriers of neuropathic TTR mutants, but not with plasma from normal individuals, thus detecting subtle structural changes that occur in amyloidogenic TTR tetramers [7].

To identify the possible existence of altered TTR conformations/modifications in tissues and plasma of FAP individuals, we produced several monoclonal antibodies against the Y78F mutant. In the present report, we characterize a particular mab designated by AD7 that detects a glycosylated form of TTR in the plasma of V30M carriers not present in normal plasma.

## Materials and methods

### Human samples

Plasma samples from asymptomatic (*n* = 180), symptomatic heterozygotic (*n* = 12) and homozygotic (*n* = 3) V30M carriers, as demonstrated by DNA analysis and their non-carrier individuals (*n* = 214) were obtained either from the Center for Predictive siblings and Preventive Genetics at IBMC, or different hospitals, all with informed consent, following the Declaration of Helsinki.

Plasma samples from individuals who had undergone domino liver transplantation (DLT), hosting the liver of V30M FAP patients (*n* = 2) were subsequently collected at different times up to 1 year, ranging from 1 to 2, 10,20,30,45, 60, 120 and 180 days.

Plasma from transplanted V30M patients (*n* = 6) was collected 24 hrs and on the followings days, as well as several months after transplantation; both procedures were approved by the Ethics Committee of the Transplantation Department, University Hospitals of Coimbra, Portugal and included informed consent.

As described throughout the results section, plasma samples from carriers of different published TTR mutations were available in the laboratory and had been collected after informed consent.

Kidney autopsy tissue from a FAP patient was available at Hospital Geral de Santo António, Porto, Portugal.

### Mice samples

Thirty plasma samples, nine cerebrospinal fluid samples - CSF (age between 6 and 18 months) and intestine (6-months old) from transgenic mice for the human V30M mutation in a TTR null background [8] collected upon sacrifice (6 months) were analysed. Mice used in the immunization protocol were 16-months old TTR null mice [9]. Mice were housed in pathogen-free conditions in a controlled-temperature room and were maintained under a 12-hrs light/dark period. Water and food were freely available. All experiments were performed in accordance with the European Communities Council Directive (2010/63).

### Mice immunization and fusion protocol

Mice (*n* = 3) were immunized with recombinant mutant TTR Y78F; the best responder animal exhibiting positive results for TTR recognition by direct ELISA was killed, blood collected and spleen removed to perform fusions, following standard procedures and multiple clones were then screened by sandwich ELISA for positive IgG to human plasma from carriers of the V30M mutation, but not to normal human plasma and then re-cloned by serial dilution.

### Mab isotyping

Microtitre plates (96 wells, Nunc) were coated with rabbit antimouse IgG – Fc specific. After blocking, plates were incubated with 100 μl/well of undiluted serum-free hybridoma culture supernatant, and then isotyped for IgG1, IgG2a, IgG2b and IgG3 classes. (AbD, Serotec). Bound antibodies were detected with streptavidin-biotinylated horseradish peroxidase (GE Healthcare).

### Enzyme-linked immunosorbent assay (ELISA)

In a direct approach, microtitre plates (96 wells, Nunc) were coated with 1 μg of isolated plasma TTR or 100 μl of serum diluted 1:10 in coating buffer. Following blocking, plates were incubated with undiluted hybridoma culture supernatants and then with sheep antimouse immunoglobulins G-HRP conjugated (Pierce) (1:5000 dilution in PBST). Plates were developed using 5 mM 2.2′-azinobis (3-ethybenzthiazoline-6-sulfonic acid) (ABTS) (Sigma), and measured at 405 nm.

In a ‘sandwich approach’, plates were first incubated at 4°C with rabbit anti-TTR polyclonal antibody (DAKO) in a 1:500 dilution in coating buffer. About 5 to 100 μg or 100 μl of diluted serum (1:10) were applied and incubated with undiluted hybridoma culture supernatant, for 1 hr at RT. After washing, bound antibodies were detected as described above.

### Mab purification

Isolation of the mab from cell culture supernatants was carried out on a Protein G Sepharose High Performance column (MabTrap G II protein G – GE Healthcare). The mab was eluted from the affinity column with Immuno Pure Gentle Elution Buffer (Pierce).

### TTR purification

Small quantities of plasma TTR were purified by immunoaffinity chromatography by cross-linking (with dimethyl pimelimidate) 8 mg of purified antibody to 1 ml Protein G Sepharose. To obtain larger quantities of purified plasma TTR to use in clone screening, and immunization, TTR was isolated by ion exchange chromatography and preparative electrophoresis [10]. Y78F TTR was produced in an Escherichia coli expression system, isolated, and purified as described [4].

### Immunoprecipitation of TTR

Five micrograms of purified mab pre-adsorbed to 50 μl of Protein G Sepharose was mixed with 25 μl of human plasma from healthy individuals or V30M carriers, overnight at 4°C, centrifuged and the pellet analysed by 15% acrylamide SDS-PAGE. TTR bands were visualized after immunoblotting using the mab.

### Fibril extraction

The Kaplan method [11] was applied to extract human TTR amyloid fibril from FAP kidney and/or TTR aggregates from the intestine of transgenic mice for human V30 M.

### Gel electrophoresis and immunoblotting

Proteins were analysed either in native or denaturing conditions. Native electrophoresis was carried out on 10% (w/v) acrylamide gels. Electrophoresis under denaturing conditions was performed in SDS-PAGE gels (15% acrylamide, 0.1% (w/v) SDS), after heat treatment of samples and addition of 0.1 M β-mercaptoethanol. Proteins were transferred from gels into nitrocellulose membranes (Hybond™-C pure, GE Healthcare), using a Tris-Glycine system, for 1 hr. After blocking and washing with PBST, immunodetection was performed with the mab in pure hybridoma supernatant, for 1 hr at RT, and sheep antimouse immunoglobulins-HRP conjugated (Pierce, 1:5000 dilution). TTR was visualized using either the enhanced chemiluminescence method (ECL, GE Healthcare) or 3,3′-Diaminobenzidine (DAB) substrate.

### Mass spectroscopic analyses and N-terminal sequencing of TTR

MALDI mass spectroscopic analysis of TTR was performed after band excision from a silver stained gel and digestion with trypsin followed by identification of the originated peptides from the SWISSPROT database; N-terminal sequencing was carried out after transfer of the proteins of interest from a SDS gel to a PVDF membrane and Coomassie staining.

### Deglycosylation

Approximately 2 μl of whole plasma were treated with 5 units of N-glycosidase F by a denaturing standard protocol according to the supplier (Calbiochem) followed by immunoblotting analysis with the mab. Five microlitres of plasma of homozygotic V30M plasma were treated with 25 units of N-glycosidase F by the same non-denaturing standard protocol and analysed by a direct ELISA procedure using the mab, as above described.

### Tunicamycin treatment

Approximately 3 × 10^5^ cells of a hepatoma cell line – SaHep [12] were stably transduced with a CSCW2 lentiviral vector to express the human wild-type and the V30M TTR variant. Cells cultured in MEM, supplemented with 10% foetal bovine serum, 2 mM glutamine (all materials from Gibco, Invitrogen) and 1 mM of non-essential amino acids (Sigma) were maintained at 37°C in a 5% CO_2_ humidified atmosphere. Cells were washed and medium without FBS was added. One flask was used as control and 1 μg/ml of tunicamycin (Sigma) added to the other flask. After 16 hrs incubation, supernatants were recovered for further analysis. About 100 μg of protein from conditioned media without tunicamycin treatment were analysed by immunoblotting with the mab as described above. Concentration of secreted TTR was assessed by ELISA by an in house-assay.

All data were expressed as mean ± SE. D'Agostino and Pearson tests evaluated normal distributions. The values of treated cells were expressed as percentage relative to controls. The differences were analysed by one-way analysis of variance (anova) with Tukey's *post hoc* tests. GraphPad Prism version 5.04 for Windows (GraphPad Software, San Diego California USA, http://www.graphpad.com) was employed.

## Results

### Screening

Following fusion, clones were screened for antibody production by testing the culture supernatants in a sandwich ELISA assay that selectively detects antibodies recognizing native antigens. Selected clones recognized: (i) recombinant Y78F TTR; (ii) purified V30M TTR from the plasma of heterozygotic carriers, but not TTR purified from normal plasma; (iii) TTR from the plasma of heterozygotic V30M carriers, but not from normal individuals.

One stable hybridoma, named AD7F6, thereafter referred to as AD7, of the IgG2b isotype was positive for the three above described criteria.

### AD7 discrimination of V30M plasma

#### ELISA

The discriminative properties of AD7 were further studied by sandwich ELISA in different carriers and conditions. Discrimination of V30M *versus* normal plasma was confirmed in heterozygotic V30M patients (*n* = 12), in homozygous V30M patients. (*n* = 3). The intensity of the reaction did not correlate between hetero and homozygotic patients, suggesting that under the conditions tested, AD7 is of value only in qualitative tests. We then tested plasma from asymptomatic V30M carriers (*n* = 200) and observed positive immunoreactivity as compared with non-carriers (*n* = 180). Samples were considered positive after subtraction of OD values from three negative controls tested on the same ELISA plate.

We then analysed plasma from V30M patients who had undergone liver transplantation and found that the ELISA values resembled those found for the normal individuals. Moreover, the ELISA levels in the plasma of normal recipients of livers from V30M carriers after domino transplantation were similar to those found for carriers of the V30M mutation, even after 1 day post transplant.

Further testing by ELISA of plasma from carriers of non-amyloidogenic TTR mutations, such as G6S (*n* = 5) and T119M (*n* = 2), or from patients with senile systemic amyloidosis (SAA; *n* = 4) showed no reactivity. Finally, we conducted a limited study on the reactivity of AD7 for plasma of carriers of amyloidogenic mutations including G49A, S50R and F64L (*n* = 3, each) and although the two first mutants were positive, carriers of F64L behaved as control individuals. Plasma from carriers of other amyloidogenic TTR mutations, namely G47A and T59K were also recognized by the mab, but these results must be confirmed because only one sample of each of these mutants was available for analysis.

Using a direct ELISA protocol, the mab maintained the discriminative properties between carriers of the V30M, G49A and S50R mutations over controls or carriers of non-amyloidogenic mutations. Carriers of the F33V, V30A and K70N mutants (*n* = 1 each) that showed negative reactivity by AD7 by the sandwich ELISA protocol reacted in a positive fashion by the direct protocol, as compared with normal plasma, probably due to exposition of cryptic epitopes in the direct ELISA approach (binding to the plastic).

#### Immunoblotting and immunoprecipitation

**Fig. 1:**
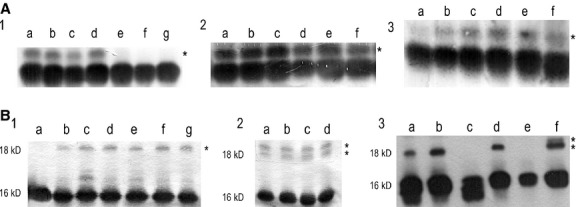
(**A**) Immunoblotting analysis of plasma TTR after native PAGE electrophoresis. The SMT - TTR band (*) was detected in: (1) plasma from TTR V30M carriers (a–d) and absent in negative controls (e–g); (2) plasma from transgenic mice for V30M TTR (a–g); (3) plasma from domino recipients, collected at different time-points after transplantation (day 1 – lane a; days 10,20,30 and 45 – lanes b,c,d and e respectively), and 4 months (lane f). (**B**) Immunoblotting analysis of plasma TTR after SDS-PAGE electrophoresis. The SMT - TTR band (*) was detected in: (1) human plasma from TTR V30M carriers (b–g) and is absent in the negative control of lane a; (2) transgenic mice for V30M TTR (a–d); (3) immunoprecipitates of human V30M plasma (a,b and d) absent in control plasmas (c and e).

Plasma from asymptomatic and symptomatic V30M carriers (*n* = 25 and 12 respectively) were separated by both native and SDS-PAGE and blotted into a nitrocellulose filter which was probed with the mab. As documented in Figure 1, besides the regular TTR band, a slow migrating TTR band (SMT, labelled with an asterisk) was readily detected in the plasma of V30 M carriers under both conditions; this band was absent in non-carrier controls (*n* = 25) (immunoblot 1 in Fig. 1A for native PAGE and immunoblot 1 in Fig. 1B for denaturing PAGE conditions respectively). This SMT band was also detected in immunoblot analyses (from native and denaturing gels) of plasma from transgenic mice for the human V30M (*n* = 25) (immunoblots 2 of Figs. 1A and B). A doublet SMT band was observed under denaturing conditions; however, it was not observed in CSF samples of these animals (*n* = 4 pools of 3 animals) (not shown). Furthermore, the SMT band was observed in the blots of plasma from recipients of domino FAP livers collected at different time-points, starting at day 1, as shown in blot 3 of Figure 1A. On the other hand, SMT was not visible on the blots of sera of transplanted V30M FAP patients collected right after 24 hrs after transplant and on the following days as well as several months (not shown).

SMT was also observed in double V30M/T119M compound heterozygotes; it was absent in heterozygotic carriers of non-amyloidogenic TTR mutations included in the study, i.e. G6S and T119M. Preliminary analyses of limited samples of carriers of non-V30M mutations (as above described in 3.2.) showed the SMT band in K70N and V30A carriers. The intensity of SMT correlated with the amount of TTR monomer with the regular migration, representing approximately 0.2% and was similar between hetero-and homozygotic V30M carriers either asymptomatic or symptomatic. We then searched for the SMT band in blots of TTR V30M aggregate/fibrils extracts of human and mice tissue origin. The SMT band was not detected in these samples (not shown).

Finally, plasma from V30M carriers and normal controls was immunoprecipitated with the mab and the immunoprecipitates analysed by immunoblots using the mab. As seen in panel 3 of Figure 1B, SMT was immunoprecipitable only from carriers of V30M and absent from controls, corroborating the immunoblot analyses performed on direct plasma. Similarly, after immunoprecipitation of plasma from transgenic mice for human V30M, two SMT bands were visualized.

### Identification of SMT

Plasma of V30M carriers was subjected to affinity chromatography using the immobilized mab, the proteins in the eluate separated by SDS-PAGE and both the silver-stained SMT and normal TTR monomer bands used for characterization. Mass spectrometry of SMT after enzymatic digestion revealed peptides with the expected masses for digested TTR (except for the first 16 residues not detected on the analyses); both normal peptide entailing residues 16–35 and the mutant peptide with a mass shift corresponding to a substitution of methionine for valine were detected as well as their oxidation products as previously reported for TTR [13]. The results obtained for the regular TTR monomer were similar, i.e. both the regular monomer and the SMT bands contained mutated and non-mutated TTR forms. From the N-terminal analyses of the SMT band, we concluded that the N-terminal was intact.

All together the results were suggestive of a post-translational modification occurring in TTR in V30M liver, but not in normal liver, which is then secreted in plasma.

**Fig. 2:**
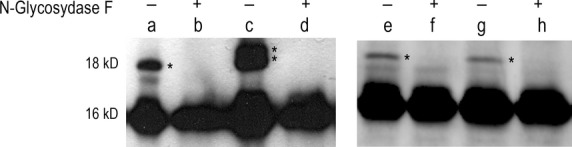
Immunoblotting after deglycosylation with N-glycosidase F. Human plasma from V30M TTR carriers before (a) and after (b) deglycosylation and from transgenic mouse before (c) and after N-glycosidase treatment (d). Human plasma from a carrier of a double mutant V30M/T119M before (e and g) and after deglycosylation (f and h). In all samples, SMT (*) is no longer detected after enzymatic removal of N-glycans.

We reasoned that the SMT recognized by the mab might represent glycosylation, considering the approximately 2000 kD increment relative to the regular monomer and the heterogeneity found in plasma of V30M transgenic mice; therefore, plasma from V30M carriers and double heterozygotic V30M/T119M or from V30M transgenic mice were treated with N-glycosidase F and analysed by immunoblot using the mab, before and after treatment. As seen in Figure 2, after N-glycosidase F treatment, SMT was no longer detected by the mab after enzymatic removal of N-glycans. We conclude that SMT represents a N-glycosylated form of TTR, secreted from livers producing the V30M mutation both in humans and transgenic mice. SMT doublet band from transgenic mice is thus possibly related to sugar heterogeneity.

We finally investigated whether the discriminative properties of the mab for V30M carriers *versus* non-carriers on the ELISA procedure could relate to V30M glycosylation. With this purpose, we treated V30M homozygotic plasma with N-glycosidase F and compared the mab reactivity by sandwich ELISA in untreated *versus* untreated samples. No change in immunoreactivity was found.

### V30M TTR secretion by hepatomas

We next searched by immunoblotting for SMT in the secretion media of a human hepatoma cell line that does not produce/secrete endogenous TTR [12] after infection with lentiviral vector stocks that lead to the production and secretion of WT and V30M TTR. SMT was not detected in either case, in contrast with the *in vivo* situation, despite of a high concentration of the regular monomer (not shown). The same conclusions were reached in parallel experiments with a HEK cell line.

**Fig. 3:**
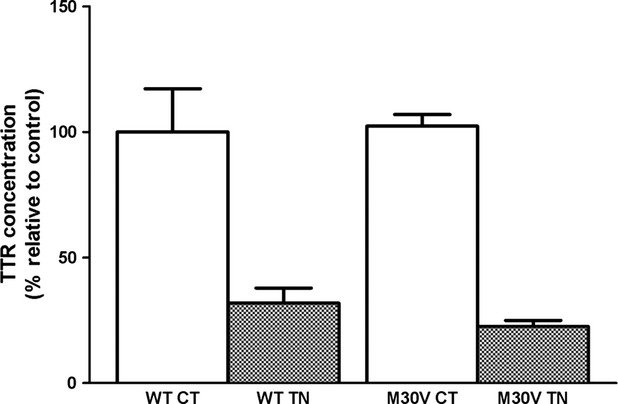
Effect of tunicamycin (TN) on secretion of TTR from WT and V30M lentiviral infected SaHep cells. Data were expressed as relative percentage to control (untreated) of tunicamycin treated cells, from five independent experiments.

Under the experimental conditions, the concentration of secreted WT or V30M TTR in the media averaged 5 μg/ml/10^5^ cells; when incubated with tunicamycin, the relative percentage levels of secreted TTR in treated *versus* untreated conditions was not significantly different between WT and V30 M producing cells (Fig. 3), which corroborates the discrepancy in the observation of N-glycosylation found *in vitro* and *in vivo* observations.

## Discussion

The presented mab has unique properties as it is immunoreactive under native conditions of ELISA of human plasma from V30M carriers or recipient individuals that received a V30M liver. It is not reactive with plasma from normal individuals, transplanted V30M FAP patients that received a normal liver or from carriers of two tested non-amyloidogenic mutations. Other amyloidogenic mutations were also immunoreactive with the mab, but the limitation of the number of samples under test does not allow us to conclude on whether the mab properties are specific for the V30M mutant where we have solid evidence.

The most significant finding was the identification by the mab of the SMT band in the plasma of the very same carriers of V30M tested by ELISA whether derived from genetic carriers or by recipients of TTR V30M liver and the disappearance of the SMT band after transplantation of a normal liver. SMT was also in plasma from transgenic V30M mice, but not in CSF. The overall data suggested the circulation of TTR conformers in the plasma of V30M carriers recognized by the mab. It should be noted that TTR is resistant to complete unfolding in SDS-PAGE, migrating as a 16,000 Kd band, above the predicted molecular mass of 14,300. The non-amyloidogenic T119M mutant did not suppress SMT in V30M/T119M allelic double compound heterozygotes. T119M TTR has been described as a non-amyloidogenic TTR variant. In compound heterozygotic individuals, the evolution of the disease seems to be more benign than in typical V30M TTR patients, suggesting a protective effect of T119M TTR on the pathogenic effects of V30M TTR. Physical–chemical studies on T119M plasma showed a higher resistance to dissociation of TTR tetramers into monomers, in contrast to a lower resistance in V30M plasma and a similar resistance to tetrameric dissociation between T119M/V30M compound heterozygotes and normal plasma [14].

Of even more interest was the identification in plasma of a N-glycosylated TTR fraction in carriers of the V30M mutations, undetectable in plasma of normal individuals. Because TTR is not a glycosylated protein, despite possessing a sequon for N-glycosylation at position 98 (Asn-Asp-Ser) the question arises what factor/s influence N-glycosylation during liver synthesis and intracellular traffic in the secretory pathway, and the disposal of N-glycosylated V30M once in circulation. Because both events contribute to the pool of N-glycosylated TTR in plasma, it is difficult to mechanistically explain both the total levels of N-glycosylated TTR, and selectivity of glycosylation. The data from the electrophoretic and mass spectrometry analyses raise a number of questions: (i) homozygotic and heterozygotic carriers have approximately the same proportion of the SMT band relative to the total monomeric unglycosylated band both under native or SDS-PAGE conditions; is this related to steric hindrance and/or different conformation between hetero- and homotetrameric V30M with consequences in glycosylation, or to a different disposal once in circulation, which could relate to differential uptake by cells or unstability ? (ii) the SMT band revealed N-glycosylation of both wt and V30M monomers; does this reflect N-glycosylation of the normal monomer in V30M heterotetramers ? The tunicamycin experiments in hepatomas corroborate the notion that wt homotetramers are retained in the Golgi with this treatment to the same extent as V30M homotetramers; it is difficult to predict the behaviour of wt/V30M or T119M/V30M heterotetramers as the V30M mutant monomer might impact on the conformation of the normal (or T119M) monomer in tetrameric hybrid species.

More than 100 different TTR mutants have been reported, although onset of the disease, tissue selectivity and severity are different among them. Earlier *in vitro* studies using HEK cells on the secretion efficiency of different mutants, including V30M, support the hypothesis that secretion differences by liver and the choroid plexus relate to protein chaperones and metabolite chaperone content of the ER in different tissues; eventually some other factors, are likely to strongly influence tissue specificity, severity, and age of onset of TTR amyloidoses [15]; most TTR mutants, such as the V30M are secreted with the similar efficiencies as WT and only most highly destabilized TTR mutants, as the case of D18G, associated with leptomeningeal amyloidosis are subjected to ER-associated degradation (ERAD); however, the precise mechanisms used in quality control to deal with the different amyloidogenic TTR mutants were not explored in this work, namely post-translational events, in particular N-glycosylation.

N-glycosylation, a highly conserved process that is characterized by the addition of a sugar complex to the nascent polypeptide chain as soon it emerges into the ER, affects protein folding, oligomerization and stability [16]. Oligosaccharyl transferase (OST) catalyses the transfer of the oligosaccharide to the asparagine side chain of the acceptor polypeptide into a consensus sequence Asn-Xaa-Ser/Thr.

N-glycosylation is normally a cotranslational process, but in 2005 Bolt *et al*. [17] demonstrated for the first time post-translational N-glycosylation in human coagulation factor VII, a non-modified protein in mammalian cells raising the question about how many glycoproteins that undergo post-translational N-glycosylation can be a part of natural processing. In 2009, Ruiz-Canada *et al*.[18] reported that oligosaccharyl transferase isoform STT3B efficiently mediates post-translational N-glycosylation when a sequon is found close the C-terminus of an unfolded protein. They proposed that nascent polypeptides meet multiple OST complexes as they emerge in the ER lumen to provide proteins proper glycosylation and folding before they leave the secretory pathway. Recently, seminal extensive studies by Sato *et al*. [19] hypothesized STT3B-dependent post-translational N-glycosylation as part of a triage-salvage system recognizing cryptic N-glycosylation sites of secretory proteins to preserve protein homeostasis. This STT3B-dependent alternative pathway for degradation is EDEM3-mediated N-glycan-dependent ERAD, distinct from the major pathway of Herp-mediated N-glycan-independent ERAD. These authors used TTR mutants in their studies to conclude that prolonged TTR unfolding in the ER occurs with highly amyloidogenic TTR mutants, as the D18G mutation and induces cryptic N-glycosylation of asparagine side chain at position 98 of TTR, a process dependent of STT3B. This conclusion emerged from genetic studies with D18G TTR and other TTR mutations (V30M included) transfected in HEK cells; in their studies V30M behaved as WT-TTR without evidence for N-glycosylation. In the present *in vitro* studies in human hepatomas secreting WT and V30M, we did not detect glycosylated TTR in the media, neither did tunicamycin affect secretion efficiency. Do the present *in vivo* findings underlie escape from ERAD systems and improved secretion of the TTR variant? Why liver secretes glycosylated V30M into plasma, but choroid plexus does not, needs investigation to dissect further unknown mechanisms in the surveillance system for protein secretion that most likely are cell specific.

At any rate, the absence of glycosylated TTR species in extracts of material from tissues with TTR deposition not specialized in TTR synthesis might relate to degradation events associated with instability of circulating N-glycosylated species or differential uptake by cells. Future studies will determine the impact of plasma N-glycosylated TTR in the etiopathogenesis of FAP and should identify the sugar moiety, and the glycosylated peptide.
